# COVID-19 in Immunosuppressed Children

**DOI:** 10.3389/fped.2021.629240

**Published:** 2021-04-29

**Authors:** Emanuele Nicastro, Lucio Verdoni, Laura Rachele Bettini, Giovanna Zuin, Adriana Balduzzi, Giovanni Montini, Andrea Biondi, Lorenzo D'Antiga

**Affiliations:** ^1^Pediatric Hepatology, Gastroenterology and Transplantation Unit, Hospital Papa Giovanni XXIII, Bergamo, Italy; ^2^Pediatric Unit, Hospital Papa Giovanni XXIII, Bergamo, Italy; ^3^MBBM Foundation, Pediatric Department, Hospital San Gerardo, University of Milano Bicocca, Monza, Italy; ^4^Pediatric Nephrology, Dialysis and Transplant Unit, Fondazione Istituto di Ricovero e Cura a Carattere Scientifico Ca' Granda, Ospedale Maggiore Policlinico di Milano, Milan, Italy

**Keywords:** SARS–CoV−2, kidney transplant, rheumatologic diseases, liver transplant, cancer, immunosuppression, autoimmune disease, innate immunity

## Abstract

Following the spread of the SARS-CoV-2 infection and coronavirus disease 2019 (COVID-19) to a global pandemic, concerns have arisen for the disease impact in at-risk populations, especially in immunocompromised hosts. On the other hand, clinical studies have clarified that the COVID-19 clinical burden is mostly due to over-inflammation and immune-mediated multiorgan injury. This has led to downsizing the role of immunosuppression as a determinant of outcome, and early reports confirm the hypothesis that patients undergoing immunosuppressive treatments do not have an increased risk of severe COVID-19 with respect to the general population. Intriguingly, SARS-CoV-2 natural reservoirs, such as bats and mice, have evolved mechanisms of tolerance involving selection of genes optimizing viral clearance through interferon type I and III responses and also dampening inflammasome response and cytokine expression. Children exhibit resistance to COVID-19 severe manifestations, and age-related features in innate and adaptive response possibly explaining this difference are discussed. A competent recognition by the innate immune system and controlled pro-inflammatory signaling seem to be the pillars of an effective response and the premise for pathogen clearance in SARS-CoV-2 infection. Immunosuppression—if not associated with other elements of fragility—do not represent *per se* an obstacle to this competent/tolerant phenotype in children. Several reports confirm that children receiving immunosuppressive medications have similar clinical involvement and outcomes as the pediatric general population, indicating that maintenance treatments should not be interrupted in suspect or confirmed SARS-CoV-2 infection.

## Introduction

Following the growth of the SARS-CoV-2 outbreak to a global pandemic, great concerns have arisen worldwide that immunocompromised patients may be at high risk of developing its severe respiratory and systemic manifestation, the coronavirus disease 2019 (COVID-19). Although children are demonstrated to be protected from developing COVID-19 by an age effect, the impact of immune suppressant treatments has been suspected to negatively impact the clinical picture as in other viral infections, such as adenovirus, rhinovirus, norovirus, influenza, and respiratory syncytial virus (1).

Despite the fact that lymphopenia has emerged as a prominent feature of severe COVID-19, clear evidence that patients under immunosuppressive therapy are at a higher risk if infected has never been demonstrated in SARS-CoV-2 or in previous coronavirus (CoV) epidemics (2–5).

Conversely, the concept that COVID-19 is primarily a systemic dysregulation of inflammatory response has come to light, whereby it has been clarified that uncontrolled innate immune signaling could highjack adaptive immunity (6). This would create a detrimental inflammatory milieu, ending up promoting disease severity (7).

We aim to review the evidence about the role of immune innate and adaptive responses and their modifiers with regard to clinical outcomes of SARS-CoV-2 in immunocompromised children.

## Methods

A literature review using online database PubMed was done using the following key words: “SARS-CoV-2” OR “COVID-19” searched in association with “children,” “immunosuppression,” “immunocompromised,” “autoimmune disease,” “cancer,” “transplantation,” “innate immunity,” “adaptive immunity,” “immune tolerance,” “pediatric rheumatic diseases,” “immunosuppressive drugs,” “biologic drugs,” “chronic kidney disease,” “pediatric renal transplantation,” “chronic liver disease,” “pediatric liver transplantation,” “hematologic disorders,” and “hematopoietic stem cell transplantation.” Results were summarized according to immune mechanisms of disease and to clinical scenarios of immunosuppression in pediatric medicine. The current evidence about the impact of COVID-19 in children immunosuppressed for rheumatologic, renal, gastrointestinal, hematologic, and oncologic conditions were discussed by as many pediatric subspecialists, and considerations about the healthcare management of such patients were made.

## Results

### The Coronavirus Family: Natural Reservoir and Immune Tolerance

The CoVs are a family of enveloped, positive-sensed, single-stranded RNA viruses infecting vertebrates, discovered in the 1960s (8). Until the early 2000s, only four species had been known to infect humans as endemic causative agents of seasonal common colds: E229, OC43, NL63, and HKU1 (9–12). In 2002, SARS-CoV-1 came to the attention of the global media as the cause of the severe acute respiratory syndrome outbreak that originated in the Guangdong Chinese province, and it ended in July 2003 with a toll of ~8,000 infected and a mortality as high as 9.6% (13). Ten years later, the Middle-eastern outbreak MERS-CoV showed overlapping clinical features with SARS-CoV-1 although a mortality of 35% (14). SARS-CoV-2 is the third highly pathogenic species originating from a zoonotic spillover in the last two decades. In fact, all human CoVs have zoonotic origin: beta-CoVs (OC43 and HKU1) are originated in mice, and alpha-CoVs (E229, NL63, SARS-CoV-1, MERS-CoV, and SARS-CoV-2) come from bat reservoirs (15, 16). Intermediate hosts have been identified in some of these events, ultimately spreading the infection to the human population.

Toward the purpose of understanding the immune host–pathogen relationship, the phylogenetic perspective involving major CoVs and bats are of great interest for clinicians. The fact that bats have been harboring the ancestors of the human CoVs and have co-evolved with them for thousands of years without displaying ill effects makes these animals valuable models of viral tolerance (17). In fact, bats have evolved unique innate immune characteristics and metabolic traits that are thought to explain their control of cytokine response as well as their extraordinary longevity and physical abilities, including their unparalleled capability of powered flight among mammalians.

Bats seem to effectively recognize pathogen-associated molecular patterns and are capable of mounting robust innate and adaptive responses but exhibit reluctance to inflammation. All bat species have acquired some reinforcement of the interferon response, fostered by enhanced expression of IFN regulatory factor 7 (IRF7) and IFN-α and by the additional phosphorylation site S185 in the IFN regulatory factor 3 (IRF3) (18–20). However, comparative genome studies have documented that downstream inflammatory response is dampened through positive selection of mutation in NLRP3 and in the c-REL binding motif of the TNF-α promoter (21). The entire PHYIN gene family—implicated in virus-related DNA damage recognition—is missing in several bat species, and the bat STING protein lacks an S358 residue critical for type I IFN response enhancement (22, 23). These arrangements ultimately lead to reduced expression of TNF-α, IL-1-β, and IL-18 and increased levels of the regulatory cytokine IL-10, allowing competent innate immunity while keeping inflammatory response in check.

Taken together, these data anticipate the central role of innate defense in the pathogenesis of COVID-19 in humans.

### Innate Immunity and Clinical Aspects of COVID-19

Innate immunity is paramount in determining clinical expression of SARS-CoV-2 infection.

As in other models of infection, SARS-CoV-2 engages receptors on innate immune cells, which initiate downstream pathways triggering secretion of several signaling molecules (6). Interferon (IFN) type I/III responses—when timely achieved and properly localized—are thought to be crucial in limiting CoV infections (24). Respiratory epithelial cells, alveolar macrophages, and neutrophils recognize viral pathogen-associated molecular patterns (PAMPs)—including viral RNA itself—and damage-associated molecular patterns (DAMPs) via different receptor families, upregulating the synthesis of several cytokines and chemokines (IL-6, MCP-1, CXCL10, IFN type I and III responses) (4, 25–27).

When the IFN response becomes pathologic, it leads to increased expression of the SARS-CoV-2 receptor ACE2 in the lungs and paves the way to aberrant inflammatory response, contributing to immuno-pathogenesis (28, 29). A well-documented IFN-dependent cytokine profile marks this shift (30, 31). Enhanced expression of cell-adhesion molecules and higher cytokine content (IL-6, IL-8, and IL-1β) mediate the recruitment of the excess macrophages and neutrophils in bronchoalveolar lavage of patients with severe disease.

In this context, lymphopenia—resulting from T cell recruitment and exhaustion—has been initially identified as the most important single biomarker predictive of severe forms and fatal outcome (26).

The relevance of the innate immune response to the clinical expression of COVID-19 and the key role of IFN-I were previously observed also for SARS-CoV-1 infection (24, 27).

Further demonstration of the centrality of the innate immunity in the antiviral response comes from genomic studies. The higher disease severity and mortality of COVID-19 observed in the African American population might be explained—at least in part—by the higher expression of IL-1β, IL-18 receptor, IL-12Rβ1, and some toll-like receptors (7).

Genome-wide association studies succeeded in identifying as candidate outcome determinants a locus containing a cluster of chemokine receptors, such as XCR1, CCR9, and CXCR6 (32) and the gene SLCA20, affecting cytokine production (33).

Apart from the cytokine footprint, other components of the innate system might play a role.

Innate lymphoid CD56^bright^ and CD56^dim^ NK cells—specialized in cytokine production and cytotoxicity, respectively—are significantly depleted in SARS-CoV-1 and SARS-CoV-2 infections (34, 35).

Finally, complement activation could link the innate immune response to the multiorgan involvement of COVID-19, mediating dysregulated neutrophilia, endothelial injury, and hypercoagulability, and determining a prothrombotic environment distinct from disseminated coagulation disorder (36).

In conclusion—as demonstrated mostly in adult patients—an excess in innate immunoinflammatory responses marks severe COVID-19.

### The Role of Adaptive Immunity

Adaptive response following SARS-CoV-2 infection is less characterized. Similarly to SARS-CoV-1, lymphopenia with drastically reduced CD4+ and CD8+ T cells is the most consistent finding in moderate-to-severe COVID-19 patients, and it correlates with disease severity and mortality (37–39). B cells are decreased as well (40, 41).

Lymphopenia is mediated by the overmentioned inflammatory cytokine milieu (especially rich in IL-6, IL-10, and TNF-α) (42), via lymphocyte sequestration into lymphoid tissue and endothelia by IFN-I and TNF-α (43, 44) or by extensive cell death triggered by IL-6 and Fas-FasLigand signaling (34).

However, T and B cell function in COVID-19 are still poorly understood. In both SARS-CoV-1 and SARS-CoV-2 infections, T cell response seems not to correlate with neutralizing antibody concentrations (45), whereas specific CD8+ memory T cells exceed CD4+ memory T cells (46, 47).

In addition to their crucial role in viral clearance and long-term immunity, T cell subsets are known to participate in hyperinflammation (48), and a reduction of immunoregulatory Tregs might contribute to the phenotype in severe cases (37, 49).

As for B cell responses, it is well-known that most patients recovered from COVID-19 seroconvert within 1–2 weeks (50). The levels of the neutralizing antibodies vary, and one third of patients exhibit very low titers although as much as 20% do not have detectable antibodies (51, 52). Moreover, specific IgG tend to decrease with time, especially in asymptomatic patients, making their protective role very uncertain (51).

Notably, yet not always protective, humoral response might be detrimental in selected conditions, triggering complement or activation or antibody-dependent enhancement, the latter mediated by the engagement of Fc-receptors by non-neutralizing antibodies, a mechanism documented in SARS-CoV-1 infection (53).

### Children's Tolerance to SARS-CoV-2

From an evolutionary perspective, newborns, infants, and young children might benefit from a reduction of the susceptibility to tissue damage during infections. In fact, balanced immunopathology during infectious episodes could allow growth and development yet achieving pathogen control. Nevertheless, the concept that young children have a milder course of infections—especially viral—is well-known since long ago and is supported by several instances. When acquired vertically or in early childhood, hepatitis B and C viruses seldom cause substantial liver injury, rather usually entering a longstanding immune-tolerant phase characterized by elevated viremia and nearly normal transaminases (54–56). Similarly, herpesviruses, such as Cytomegalovirus, Epstein-Barr virus, and Varicella-Zoster virus, cause illnesses with much more limited symptoms in children compared with adolescents and adults, and this might be related to a regulated antigen presentation and to suppressed cytotoxic T cell responses (57–59). Human immunodeficiency virus-infected non-progressing children—unlike adults—exhibit high viremia but maintain CD4+ T cell counts along with low immune activity against the virus (60), a phenotype that resembles that of the simian viral homolog natural hosts (61).

Different mechanisms exist possibly explaining the viral immune tolerance in children. Neonatal physiologically enriched CD71+ extramedullary erythroid precursors have been proved to suppress innate and adaptive responses and to induce regulatory T cells via multiple soluble factors (62–64). So-called myeloid-derived suppressor cells (MDSC) help reduce mortality due to infections in infants, dampening cytokine response (65, 66), and their persistence in older children has been documented as an effect of active immunizations (67).

When it comes to explaining the mild clinical and immunopathological consequences of the SARS-CoV-2 in children, more specific mechanisms of tolerance have been elucidated.

Children have a lower ACE2 receptor expression on the nasal epithelia as compared with adults, and are probably less prone to the IFN-mediated upregulation of ACE2, thus being more resistant to SARS-CoV-2 entry (68, 69). TMPRSS2 and CD147, two other proteins assisting in viral entry, increase with age (70, 71).

Another means of resistance to SARS-CoV-2 in young patients is their lower threshold of IFN antiviral response and their higher basal expression of IFN-I/III-associated genes in the respiratory epithelium (72, 73) with respect to adults. These first traits account for a lower viral replication.

Unlike adults, children also display a much milder neutrophil (74) and monocyte/macrophage activation (75). The better functioning of phagocytosis makes unlikely their involvement in the cytokine storm as seen in the severely ill elderly.

Finally, more efficient T and B cell responses have been hypothesized in the young ones, resulting in better inflammatory outcomes and higher-quality antibody response (76, 77).

Altogether, these findings indicate that—differently from adults—children's innate responses succeed to control viral replication and achieve SARS-CoV-2 clearance without pathogenic inflammatory outcomes. Conversely, a reduced adaptive response in children vs. adults would be witnessed by a lower number of antigen-reactive CD25+ and IFN-γ-producing CD4+ T cells, quantitatively lower neutralizing antibodies, less antibody-dependent enhancement (78).

### The Evidence From Past Coronaviruses Epidemics

Besides the increasing understanding of species- and age-specific host-related immune activity against SARS-CoV-2, most of the knowledge about the immunopathology of COVID-19 gathers from related pathogens, such as SARS-CoV-1 and MERS-CoV.

SARS, MERS, and COVID-19 exhibit a common pattern of immune engagement even with some differences. SARS-CoV-2 replicates better in pulmonary tissues and presents an earlier nasopharyngeal viral load peak compared with SARS-CoV-1, whereby eliciting a less broad cytokine profile (27, 79). However, the earliest observations that SARS-CoV-1-infected patients worsened toward ARDS as their viral shedding decreased have provided a first indication of the crucial role of the exuberant immunoinflammatory host response in the pathogenesis of the disease rather than uncontrolled viral replication (80).

Beyond the important lessons on physiopathology, these past human CoV epidemics have offered substantial information to estimate the clinical burden in at-risk populations. Because dysregulated and excessive immune responses appear to be particularly important drivers of tissue damage, past epidemics may assist in testing the hypothesis that the status of an immunocompromised host might be unimportant or even protective against SARS-CoV-2 infection.

In the first SARS epidemic—which ended in July 2003 after an important public health mitigation effort—among the 8,096 subjects infected and 774 (9.6%) deaths in more than 30 countries, mortality changed according to age. In fact, the case fatality ratios were <1% in persons aged 24 years or younger, 6% in persons aged 25–44 years, 15% in persons aged 45–64 years, and >50% in persons aged 65 years and older. Household contact, male sex, and the presence of comorbidities but not immunosuppression were risk factors. Overall, 48 children under 12 years of age were diagnosed with SARS, all having only mild respiratory illness, none of them needing oxygen support (WHO about SARS). Contrary to expectations of poor outcomes in transplanted and immunosuppressed patients in case of SARS, at the end of the outbreak, no such case had been recorded (2).

Similarly, as of February 28, 2018, MERS has left behind a death toll of 779 out of 2,182 cases, mostly in Saudi Arabia. Risk factors for higher mortality were advanced age, male sex, and presence of comorbidities (obesity; diabetes; heart, lung, and kidney disease), but immunosuppressed status was not found to be a risk factor (81).

Thus, previous studies on earlier twenty-first-century CoV epidemics failed to demonstrate an augmented risk for patients undergoing immunosuppressive treatments.

### COVID-19 in Children Immunosuppressed for Rheumatologic Conditions

The increased risk of infectious complications in rheumatologic diseases can be linked both to the basic immunological dysfunction (lower production of specific immunoglobulins, low complement levels, altered phagocyte response) and to the immunosuppressive drugs used (steroids, classic, targeted, and biological disease-modifying antirheumatic drugs or DMARDs). In the pediatric field, numerous studies have investigated the susceptibility to infections of patients with juvenile idiopathic arthritis (JIA) or juvenile systemic erythematous lupus in relation to disease activity and treatment. For example, children hospitalized with JIA have a 2-fold increase in bacterial infection rates regardless of treatment and a 3-fold increased with high-dose oral steroid use (≥10 mg prednisone/day) (82). A 6–7-fold increased risk of serious infections in children treated with etanercept and adalimumab compared with methotrexate has been reported with disease activity acting as an independent risk factor as well (83). This increased susceptibility also affects viral respiratory infections that are associated with exacerbation of the underlying disease even in the absence of temporary suspension of the background therapy (84).

In March 2020, the European League Against Rheumatism-Pediatric Rheumatology European Society (EULAR-PRES) issued a recommendation against the withdrawal of immunomodulatory and immunosuppressive therapies even in absence of robust data about the impact of SARS-CoV-2 on rheumatologic disease (85).

Subsequently, different studies showed that patients on DMARDs are not at greater risk of severe SARS-CoV2 disease than the general population.

Two surveys in adult patients in northern Italy show that the incidence and severity of COVID-19 in patients treated with DMARDs are not significantly different from the rates found in the general population in the same region (86, 87). Both studies found a high adherence to the prevention measures of SARS-CoV-2 infection (social distancing, use of PPE, and handwashing), and voluntary suspension of therapy and disease relapse seldom occurred.

In the pediatric rheumatology field, a survey conducted after the first weeks of the epidemic in the hardest-hit regions in Europe (the Milan area, northern Italy) investigated patients' health conditions and history of exposure to COVID-19 (88). Out of 123 children treated with biological DMARDs (bDMARDs), associated or not with synthetic DMARDs (sDMARDs), only eight had mild respiratory symptoms, and three of them had contact with adults with suspected COVID-19. No patient interrupted the ongoing therapy, no disease relapses were reported, and all patients adhered to standard precautions. Similar results were reported in a wider Turkish series of children treated with s/bDMARDs (89). Out of 414 patients, only six suspected cases of COVID-19 were reported. The only confirmed COVID-19 case, a girl with JIA on leflunomide treatment with intrafamiliar contact history, had a rapidly favorable course with negativization of the nasopharyngeal swab 2 weeks after diagnosis. A voluntary suspension rate of DMARD therapy of 14% was found, often related to difficulty in accessing health facilities due to fear, anxiety, and/or social restrictions or to perception of risk related to the treatment. Other population surveys have reported 11–18% of mild respiratory symptoms in children with rheumatic diseases despite a high COVID-19 attack rate (90, 91).

It is possible to state that, even in children with rheumatologic disease treated with DMARDs, there is no increased incidence of SARS-CoV-2 disease or its complications. During the epidemic, it is, therefore, extremely important to keep the underlying disease under control by continuing the treatment because it is well-known that disease flares can be a risk factor for overlapping infections.

### COVID-19 in Children Immunosuppressed for Chronic Gastrointestinal Conditions

Encouraging results have been drawn in adults and children with inflammatory bowel diseases (IBD). The earliest report from a Chinese consortium of the seven largest Chinese IBD referral centers (more than 20,000 patients) reported no case of SARS-CoV-2 infection, notably not even from the three largest tertiary centers in Wuhan as of mid-March 2020 (92). The course of the epidemic was uneventful also in a cohort of 522 IBD patients (11% pediatric) from Bergamo, Lombardy, the subsequent epicenter, receiving an immunosuppressant or a biological treatment in 22 and 16% of the cases, respectively (93). A serology prevalence study on the same cohort showed a SARS-CoV-2 seroprevalence of 23% of the patients (comparable to that of the health personnel controls) although about 60% of them were asymptomatic, and the remaining had only mild symptoms without respiratory failure (94). Symptomatic SARS-CoV-2 has been described in adult IBD patients, but as reported by Taxonera et al. the overall risk of infection is milder and the case fatality rate showed no differences when comparing IBD with the general population, possibly as an effect of the substantial adherence to contact precautions (95).

Focusing on children, results from an electronic reporting system of children with IBD infected with SARS-CoV-2 among 102 pediatric IBD has identified only nine infected children worldwide (96). Notably, in Asian early affected countries, no SARS-CoV-2 infection was reported, yet delay in biologic treatment was observed in 79 children, of whom 17 (22%) had exacerbation of their IBD.

Early data about autoimmune liver diseases also came from the hardest-hit Italian province of Bergamo, Lombardy, where 148 patients were surveyed, all on immunosuppressive medications. Of 47 children (37 with autoimmune hepatitis and 11 with sclerosing cholangitis/overlap syndrome), none tested positive for COVID-19. Of the remaining adult patients, four had documented infection, and one elderly patient with comorbidities of hypertension and dyslipidemia died from COVID-19 pneumonia (97). Such data were replicated by other studies and confirmed the concept—already suggested during SARS and MERS epidemics—that patients with chronic liver autoimmune disease are not at increased risk because of immunosuppression (5, 98–100).

The disease burden is comparable in pediatric liver transplant recipients. In a survey conducted in Bergamo on 138 transplanted children residing in Lombardy, no confirmed COVID-19 infection was detected although a history of contact was present in 18 of them (which was a household contact in five), and only four developed mild symptoms but were not tested (101). Overall, some 30% of the surveyed children presented mild illness compatible with COVID-19, but none had pneumonia or required hospitalization. This scarce susceptibility replicates that of the general pediatric population and denies a role of immunosuppression as an additional risk in transplanted children. Even data from the European Liver Transplant Registry demonstrate a protective effect of the tacrolimus on the development of severe disease in adults (102).

Nevertheless, two thirds of the pediatric transplant centers have experienced a reduction in their activity as judged by a survey of the European Reference Network Transplantchild, whereas the clinical impact on solid organ-transplanted children was negligible (103).

### COVID-19 in Children Immunosuppressed for Chronic Renal Conditions

COVID-19 represents a particular challenge for patients affected by chronic renal diseases, such as end-stage kidney disease, and kidney transplantation and glomerular diseases treated with immunosuppressive therapy. These patients have a high susceptibility to infections because of malnutrition, uremia, comorbidities, and of course, the immunosuppressive treatment itself.

A number of reports from adult centers have highlighted how these types of patients frequently develop a severe form of COVID-19: during the peak of the epidemic in New York, 36 adult kidney transplant recipients tested positive for SARS-CoV-2 in 15 days at a single center (104). Most of the patients (78%) were admitted, and 28% died after a median follow-up of 21 days. This dramatic picture fortunately did not apply to the pediatric patients with chronic kidney diseases.

Overall, 36 pediatric COVID-19 cases with chronic kidney disease have been reported so far, 28 of them under immunosuppressive therapy, including 15 kidney transplant recipients. All had mild illness, and none required oxygen administration (105–107). Only 18 cases were reported from a large survey comprising 16 pediatric nephrology centers across 11 countries, indicating that the incidence of symptomatic SARS-CoV-2 infection is as low as in the pediatric general population (105).

Interestingly, in two patients with steroid-dependent nephrotic syndrome from a Spanish cohort, COVID-19 triggered a disease relapse (106). Conversely, none of the 127 children with nephrotic syndrome who had been treated with anti-CD20 chronic immunosuppression at a median time of 18 months since last infusion reported clinical symptoms for COVID-19. Six patients had cohabitants with confirmed COVID-19 (two households died), yet only one patient was tested and resulted negative (108).

Due to the uncertainty regarding the risks for immunosuppressed children, during the peak of the pandemic, the Italian Society of Pediatric Nephrology conducted a nationwide observational study (109), whose aim was to identify clinically relevant (death, admission to an intensive care unit, need for mechanical ventilation or change of the ongoing immunosuppressive treatment) COVID-19 cases. Almost 70% of the Italian pediatric population with chronic kidney diseases (1,572 children) was reached, most of them on immunosuppressive treatment. The results of our study were very encouraging as no patient fulfilled the criteria for the presence of severe COVID-19. Only three patients tested positive for SARS-CoV-2, two had symptoms (fever and skin rash), and the other was asymptomatic (109).

### Immunosuppression in Children Treated for Hematologic and Oncologic Conditions

Data regarding the clinical manifestations of COVID-19 in pediatric hemato-oncological patients are still relatively scarce (110–112).

In Lombardy—the hardest-hit Italian region—six pediatric hemato-oncological centers collected 21 COVID-19 cases during the first 8 weeks (February 20 to April 15, 2020) of the pandemic, of which 15 were patients with ongoing treatment/immunosuppression and five were in follow-up after elective treatment discontinuation. Only two patients developed pneumonia, and one of them required respiratory support (113).

An early brief survey published by Hrusak and colleagues reported only nine confirmed SARS-CoV-2 infections out of a population of almost 10,000 children and young adult patients on anticancer therapies from 25 different countries. Most of the cases had a mild course of the disease, suggesting that preventive measures should not delay oncological treatment (114).

Overlapping data and conclusions are reported in the UK national whole population-based registry of pediatric cancer patients with confirmed SARS-CoV-2: among 54 reported patients, the majority were asymptomatic (28%) or had a mild course of the disease (63%), and no major delays in cancer care were observed (115).

Nonetheless a recent large multicenter retrospective study performed in the New York and New Jersey region observed that, despite pediatric cancer patients presenting an overall low morbidity and mortality related to SARS-CoV2 infection, the risk of severe COVID-19 infection was higher compared with the general pediatric population. Specifically, the authors describe a cohort of 98 positive patients: among them, 28 required hospitalization, 25 required oxygen support, and seven required mechanical ventilation. No deaths, however, were strictly related to the infection (116). In general, the relatively small size of the cancer pediatric population does not allow assessment of whether being affected by a hemato-oncological disease is a risk factor for a severe pattern in case of exposure to SARS-CoV-2 (117). If the underlying disease has any impact, it might be counterbalanced by the protective effect of the young age as ultimately shown by the mild or asymptomatic infection of most reports (118–122).

On the other hand, as for the rate of infection, it is likely that children with hemato-oncological conditions bear the same risk of SARS-CoV-2 as the general pediatric population, but they are less frequently exposed due to the social distancing that they observe regardless of the regulated lockdown (123). In fact, out of 465 pediatric cancer patients tested for SARS-CoV-2 at the MSKCC, 34 (7.3%) were positive with no COVID-related deaths. Only four of the first 20 positive patients required hospitalization (124, 125).

Unlike children, most reports on adults agree on the higher risk of a severe COVID-19 pattern in patients with cancer. An overall 3.61-fold higher risk of severe COVID-19 pattern was reported in cancer patients compared with patients without cancer (126). Conversely—among cancer patients—a 2.45-fold increased risk of death was reported in COVID-19 affected vs. non-affected adults (127). As for patients with hematological malignancies, they seem to have 2-fold higher mortality due to COVID-19 in comparison with the non-cancer general population (128, 129).

However, whether this frailty is related to immunosuppression or to other factors is unclear. Strikingly, an older age and previous treatment with immune checkpoint inhibitors (ICIs) but not chemotherapy itself were predictors of severe disease in a large cohort from the Memorial Sloan Kettering Cancer Center (MSKCC) (130). Because ICIs aim to trigger an immune response against cancer, authors claim that immune upregulation by T cell hyperactivation, rather than immunosuppression, might facilitate lung injury, and ARDS.

In conclusion, pediatric cancer patients have overall good COVID-19 outcomes, but they are still slightly worse than the general population. In contrast, the higher risk of severe COVID-19 exhibited by adults could be explained at least in part by other organ toxicities induced by chemo/radiotherapy and risks of additional infections due to pancytopenia rather than the loss of immune competence.

### Loss of Tolerance Toward SARS-CoV-2: The Paradox of Kawasaki Disease Spectrum/MIS-C

The concept that SARS-CoV-2-infected children have an invariable mild course has been challenged by the reported occurrence of a complication occurring 4–6 weeks after infection with high fever, organ dysfunction, and strongly elevated markers of inflammation (131–134). Nearly all the children presented symptoms such as conjunctivitis, lymphadenopathy, mucocutaneous rash, and coronary artery dilation and, in the most severe cases, cardiovascular shock, encephalitis, and multiple organ failure, resembling Kawasaki disease (KD), and fulfilling its diagnostic criteria. This entity has been defined COVID-19 Associated Multisystem Inflammatory Syndrome in Children (MIS-C).

A putative RNA virus had been postulated as causative of KD long before the COVID-19 pandemic on the basis of (i) RNA virus-like inclusion bodies in respiratory epithelia of cells of KD patients and (ii) the transcriptomic signature of INF-I response—classically involved in antiviral processes—in coronary arteries of KD patients (135). A higher rate of human CoV E229-antibody positivity in children with KD seems to support that this RNA virus could be coronavirus (136). The current evidence of SARS-CoV-2 as the etiologic agent fits well the acknowledged pathophysiology of KD. In this model, PAMP recognition by the cytosolic and soluble PRRs elicit immune cell activation (137), and a plausible superantigen-like sequence motif in the spike protein induce non-specific T cell proliferation (138), both mechanisms ultimately leading to cytokine storm.

The clinical phenotype might be influenced by the patient's age as a consequence of the pandemic scenario due to a novel pathogen, explaining at least in part the unique characteristics of the so-called MIS-C (pandemic KD) with respect to the classical (seasonal) KD (139).

Even if close on clinical grounds, seasonal KD and pandemic MIS-C exhibit some differences that allow their classification into separate entities. MIS-C occurs in between 0.011 and 0.31% of children with SARS-CoV-2 infection (140). MIS-C occurs in older children; has more frequent systemic, myocardial, and gastrointestinal involvement; and complicates with shock more commonly than KD (141).

At immunological characterization, MIS-C is very different from severe adult COVID-19 and more similar to KD even with some peculiarities. Based on these data, it seems that patients with KD showed higher IL-17A levels than MIS-C and SARS-CoV-2 patients (139). However, comparable IL-17A and IFN-γ between non-severe pediatric cases and MIS-C might suggest that, in MIS-C, antiviral innate responses, and viral clearance are conserved, but in the late postinfectious phase, a distinct immune-inflammatory imbalance occurs (78).

**Figure 1:**
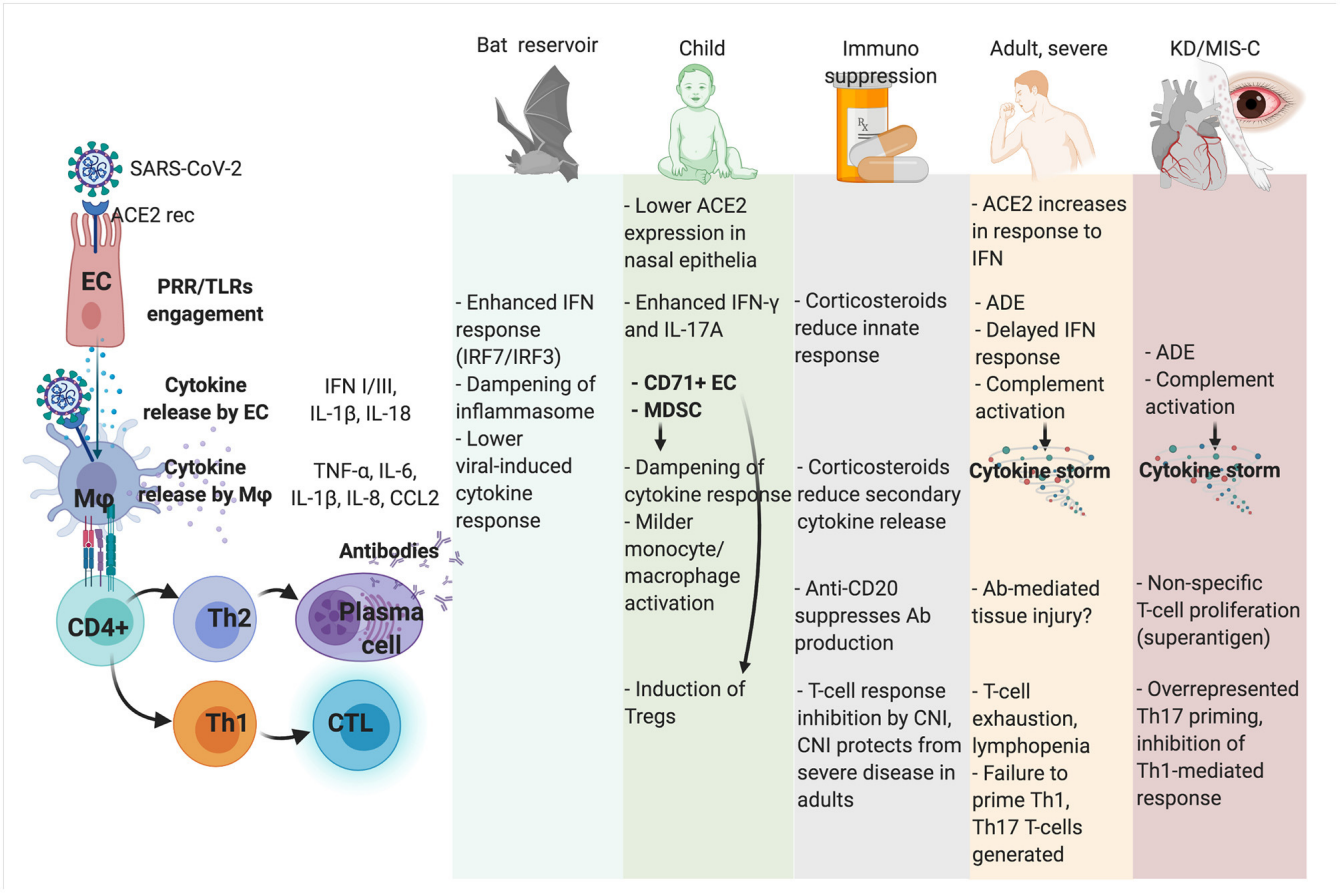
Mechanism of immune competence, tolerance, and immunopathology toward SARS-CoV-2 infection in different hosts and clinical scenarios. ADE, antibody-dependent enhancement; CNI, calcineurin inhibitors; CTL, cytotoxic T cell; CD71+ EC, CD71+ erythroid precursors; EC, lung epithelial cell; KD, Kawasaki disease; MDSC, myeloid-derived suppressor cells; Mϕ, macrophage; MIS-C, Multisystem Inflammatory Syndrome in Children; PRR, Pattern Recognition Receptor; TLR, Toll-like receptors.

In Figure 1, possible determinants of immune tolerance and immunopathology are represented in human hosts and natural reservoirs.

### Considerations for Healthcare Management in Immunosuppressed Children

In summary, the immunopathogenesis of COVID-19 indicates that immune innate and adaptive response—rather than direct virus-induced damage—are the main actors of pulmonary and extrapulmonary inflammation and tissue injury (6, 7).

In this perspective, immunosuppression might be not detrimental or even advantageous in SARS-CoV-2 infection (2, 5). Adults with autoimmune conditions seem not to be at higher risk of a severe course of COVID-19 because of immunosuppression, and in more complex conditions—such as cancer patients—the higher severity could be explained better by the overall fragility and multiorgan involvement rather than by immunosuppressed status. In addition, a clear detrimental effect of the anticancer immunotherapy has been demonstrated in oncologic adult patients (130).

Nevertheless, weighting the impact of a suppressed immunity in pediatric SARS-CoV-2 infection is far from being a simple task because children are *per se* immune tolerant to the pathogen.

In a trial in which the virus is suspected to be the instigator and the immune system the hitman, immunosuppressive treatments could be pronounced not guilty of worsening the course of COVID-19, at least in children.

According to these conclusions, immunosuppressive medications should not be withdrawn and scheduled therapies should not be delayed in such children. Also, relevant scientific societies recommend in favor of the maintenance of standard care for children needing immunosuppression for diverse conditions (85, 123, 142, 143).

## Author Contributions

EN: article design, data collection, manuscript preparation, and figure preparation. LV: data collection and manuscript preparation. LRB: data collection and manuscript preparation. GZ: manuscript revision. ABa and GM: data collection and manuscript preparation. ABi: manuscript revision. LD'A: article design and manuscript revision. All authors contributed to the article and approved the submitted version.

## Conflict of Interest

The authors declare that the research was conducted in the absence of any commercial or financial relationships that could be construed as a potential conflict of interest.
